# Environmental Contamination of SARS-CoV-2 in a Non-Healthcare Setting

**DOI:** 10.3390/ijerph18010117

**Published:** 2020-12-26

**Authors:** Judith Chui Ching Wong, Hapuarachchige Chanditha Hapuarachchi, Sathish Arivalan, Wei Ping Tien, Carmen Koo, Diyar Mailepessov, Marcella Kong, Mohammad Nazeem, Merrill Lim, Lee Ching Ng

**Affiliations:** 1 Environmental Health Institute, National Environmental Agency, 11, Biopolis Way, Helios Block, #06-05/08, Singapore 138667, Singapore; judith_wong@nea.gov.sg (J.C.C.W.); Chanditha_hapuarachchi@nea.gov.sg (H.C.H.); sathish_arivalan@nea.gov.sg (S.A.); Tien_wei_ping@nea.gov.sg (W.P.T.); carmen_koo@nea.gov.sg (C.K.); diyar_mailepessov@nea.gov.sg (D.M.); mkong003@e.ntu.edu.sg (M.K.); mohammad_nazeem@nea.gov.sg (M.N.); merrill_lim@nea.gov.sg (M.L.); 2 School of Biological Sciences, Nanyang Technological University, 60, Nanyang Drive, Singapore 637551, Singapore

**Keywords:** SARS-CoV-2, COVID-19, environment, surface contamination, polymerase chain reaction, fomite, transmission

## Abstract

Fomite-mediated transmission has been identified as a possible route for the spread of COVID-19 disease caused by SARS-CoV-2. In healthcare settings, environmental contamination by SARS-CoV-2 has been found in patients’ rooms and toilets. Here, we investigated environmental presence of SARS-CoV-2 in non-healthcare settings and assessed the efficacy of cleaning and disinfection in removing virus contamination. A total of 428 environmental swabs and six air samples was taken from accommodation rooms, toilets and elevators that have been used by COVID-19 cases. By using a reverse transcription polymerase chain reaction assay, we detected two SARS-CoV-2 RNA positive samples in a room where a COVID-19 patient stayed prior to diagnosis. The present study highlights the risk of fomite-mediated transmission in non-healthcare settings and the importance of surface disinfection in spaces occupied by cases. Of note, neither air-borne transmission nor surface contamination of elevators, which were transiently exposed to infected individuals, was evident among samples analyzed.

## 1. Introduction

The coronavirus disease 2019 (COVID-19) first emerged in December 2019 in the city of Wuhan in Hubei Province, China, and has since spread to more than 200 countries across the globe [1]. Recognized as a global pandemic by the World Health Organization (WHO) in March 2020 [2], the outbreak has caused over 70 million infections and 1.6 million deaths as of 19 December 2020. In Singapore, more than 58,000 COVID-19 cases have been reported as of December 2020, with local clusters of transmission occurring in a tourist shop, places of worship, business meetings, workplaces and worker dormitories, among others [3]. COVID-19 is caused by the Severe Acute Respiratory Syndrome Coronavirus 2 (SARS-CoV-2) and common symptoms include fever, productive cough, fatigue and shortness of breath [4]. Presently, there is no widespread use of vaccines or treatments available for SARS-CoV-2 infections. Practicing good personal and environmental hygiene and social distancing are used as preventive measures.

Similar to other human coronaviruses, SARS-CoV-2 is thought to spread through person-to-person contact, respiratory droplets and contact with contaminated surfaces [5]. Evidence of environmental contamination has been shown in healthcare settings, where SARS-CoV [6,7], Middle East Respiratory Syndrome Coronavirus (MERS-CoV) [8,9], and more recently, SARS-CoV-2 [10] have been found around the patients’ bed and toilet areas. Laboratory tests also demonstrate that SARS-CoV-2 can survive on surfaces for up to several days [11,12], further emphasizing the risk of fomite-mediated transmission, particularly in the healthcare environments.

As the contaminated environment presents a possible route of transmission, the National Environment Agency introduced measures to reduce the risk of fomite-mediated transmission after the onset of the first few cases in Singapore. Within one week of the diagnosis of the first imported cases, the agency issued specific guidelines for cleaning and disinfection of premises and residences exposed to SARS-CoV-2 infected individuals [13,14], as well as advisories to increase frequency of general cleaning of premises. Supervision for cleaning and disinfection procedures was also provided for non-healthcare premises.

In the present study, we assessed the extent of surface contamination and the efficacy of disinfection procedures at premises where infected individuals resided or visited. The surface swabs were taken before and after cleaning and disinfection of common high-touchpoint areas in accommodation rooms, toilets and elevators. As some reports suggest the possibility of aerosol-borne transmission [12,15], air samples were also taken from an enclosed air-conditioned room that had no mechanical ventilation. We detected two SARS-CoV-2 RNA positive surface swabs collected from a room where a SARS-CoV-2 infected person resided prior to diagnosis, highlighting the risk of fomite-mediated transmission in non-healthcare settings.

## 2. Materials and Methods

### 2.1. Environmental Samples

**Figure 1:**
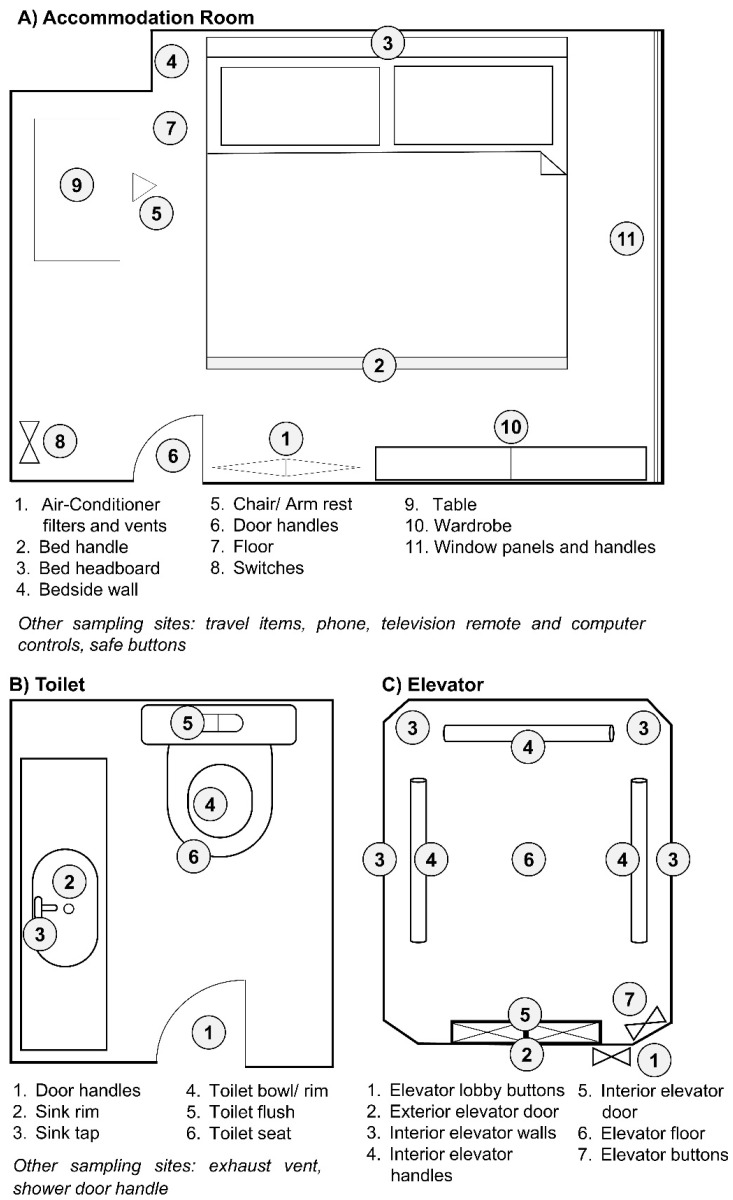
Sampling plan in each type of location. Representative sampling sites for (**A**) accommodation rooms, (**B**) toilets and (**C**) elevators. Environmental swab samples were taken at the areas indicated in numbers.

Environmental surface swabs were taken between 28 February and 20 March 2020 from common high-touchpoint areas in accommodation rooms, toilets and elevators of premises (Table 1, Figure 1). Individuals confirmed to be infected with SARS-CoV-2 have resided or visited these sites prior to diagnosis and isolation (Table 1). The accommodation rooms and toilets were from a home residence (two rooms and one toilet) and two commercial boarding residences (first location: one room and one toilet; second location: one room and six toilets), while the elevators were from two public housing blocks (three elevators per block). As diagnosis and isolation are typically conducted a few days after the onset of symptoms, it was assumed that the cases were symptomatic while residing or visiting these sites. Surfaces potentially exposed to the touch and any cough of an infected person (e.g., walls, table surfaces) were sampled.

Pre-moistened sterile synthetic-tipped swabs were used for the surface sampling. Flat and even surfaces, such as bed headboards, floor, tables and walls, were swabbed using a square template grip (10 cm × 10 cm), covering 100 cm^2^ surface area. Surfaces that are uneven or irregularly shaped, were swabbed to a maximum of an estimated 100 cm^2^ area. Field blanks were taken by moistening the swab and placing directly into the tubes with Viral Transport Media (VTM), without any surface sampling [16]. These were done at each sampling site to determine if any cross-contamination has occurred during sample collection. All swabs were placed in tubes containing 500 μL VTM after sampling [16]. Details of the sampling premises as well as the active ingredients of disinfectants used for surface disinfection are listed in Table 1.

Air samples were collected in VTM using a cyclonic air sampler (Coriolis, Bertin Instruments) with an air flow rate of 300 L/min for a duration of 30 min per sample. Air samples were collected in an accommodation room (occupied by Case 1 in Table 1) that was thought to be poorly ventilated and another two samples were collected from the external surfaces of room entrance. All samples were taken within 1–3 days after the infected persons vacated the sites and have been isolated in healthcare facilities.

### 2.2. Study/Ethics Approval

This study was approved by the Environmental Health Institute’s Management Committee (Project TS264), National Environment Agency.

### 2.3. Extraction of SARS-CoV-2 RNA from Environmental Samples

Swabs in VTM were vortexed at high speed for 60 s [17]. The wet swabs were pressed against the inner wall of tubes to squeeze out as much liquid as possible. The remaining solution was centrifuged at 13,000 rpm for 1 min to remove any debris. A 140 µL aliquot of the supernatant was used for the extraction of viral RNA by using the QIAMP Viral RNA Mini Kit (Qiagen, Hilden, Germany) according to the protocol recommended by the manufacturer. For air samples collected in VTM, 140 µL of the sample was used directly for viral RNA extraction.

### 2.4. Detection of SARS-CoV-2 RNA by Polymerase Chain Reaction

SARS-CoV-2 RNA was detected by using a conventional reverse transcription polymerase chain reaction (RT-PCR) protocol adopted from the method described by Woo et al. (2005) [18]. The oligonucleotides used in the first round of the revised nested RT-PCR protocol targeted the same genomic region of the RNA dependent RNA polymerase (RdRp) gene described by Woo et al. (2005), but were modified to be specific to SARS-CoV-2, based on sequences available in NCBI nucleotide database and GISAID’s EpiCoV™ Database [19] at the time of writing. The oligonucleotides used in the second round of PCR were designed internal to the first-round amplicon. The details of the oligonucleotides are given in Table 2.

Complementary DNA (cDNA) was synthesized from extracted RNA using random hexamers provided in the Maxima H Minus First Strand cDNA Synthesis Kit (Thermo Fisher Scientific, Waltham, MA, USA) according to the manufacturer’s instructions. Targeted regions of the first and second rounds of PCR were amplified using 0.5 µM of each primer pair (Table 2), 2 µL of the template and 1X PhusionTM Flash High-Fidelity PCR Master Mix (Thermo Fisher Scientific, Waltham, MA, USA) in a final reaction volume of 20 µL. SARS-CoV-2 synthetic RNA (Twist BioScience, South San Francisco, CA, USA) was used as the positive PCR control, whereas molecular grade water was used as the negative PCR control. The amplification protocol for both rounds was as follows: initial denaturation at 98 °C for 10 s, 35 cycles of denaturation at 98 °C for 5 s, annealing at 61 °C for 10 s, extension at 72 °C for 15 s and final extension at 72 °C for 1 min. The limit of detection of the assay was 12 copies per reaction (~6.25 copies/ cm^2^ of swabbed area). The amplified products were visualized in 1.5% agarose gels and were purified by using Expin PCR SV mini kit (GeneAll Biotechnology, Seoul, Korea) according to manufacturer’s instructions. The purified PCR products were sequenced with the respective amplicon primers at a commercial facility using the Sanger sequencing according to the BigDye Terminator Cycle Sequencing kit (Applied Biosystems, Foster City, CA, USA) protocol. Besides being highly sensitive, the nested protocol generated genomic fragments long enough to be sequenced. We also confirmed any SARS-CoV-2 positive sample by using the Taqman^®^ probe-based real-time PCR assays developed by Hong Kong University (HKU) and China Communicable Disease Centre (CDC) as per the protocols recommended by the World Health Organization [20]. The detection sensitivity of conventional nested PCR assay and probe-based real-time PCR assays were comparable (Supplementary Table S1).

### 2.5. Assembly and Analysis of Genome Sequences

Raw nucleotide sequences were assembled using the Lasergene package version 15.0 (DNASTAR Inc., Madison, WI, USA). Contiguous sequences were aligned using BioEdit 7.0.5 software suite [21] and were used in the phylogenetic analysis to compare with global sequences retrieved from the NCBI nucleotide database and GISAID’s EpiCoV™ Database [19]. The maximum likelihood tree was constructed by using the general time reversible substitution model with gamma and invariant site parameters (GTR + Gamma 5 + I), implemented in MEGA version 7 [22]. The robustness of the tree was tested by bootstrapping 1000 iterations.

### 2.6. Data Availability

Sequences of SARS-CoV-2 generated in the present study were deposited in Genbank database under the accession numbers MT309050 and MT309051.

## 3. Results and Discussion

From 28 February 2020 to 20 March 2020, 434 environmental samples comprising of 428 surface swabs and six air samples were collected across 18 sampling sites. Surface swabs were collected from four accommodation rooms (134 samples) and eight toilets (120 samples) and six elevators (174 samples), while air samples were collected from an accommodation room occupied by case 1 (Table 1 and Table 3). Half of the surface swab and air samples were taken before the cleaning and disinfection and the other half was taken after the disinfection procedure. No noticeable differences in hygiene standards were observed among the sites.

**Figure 2:**
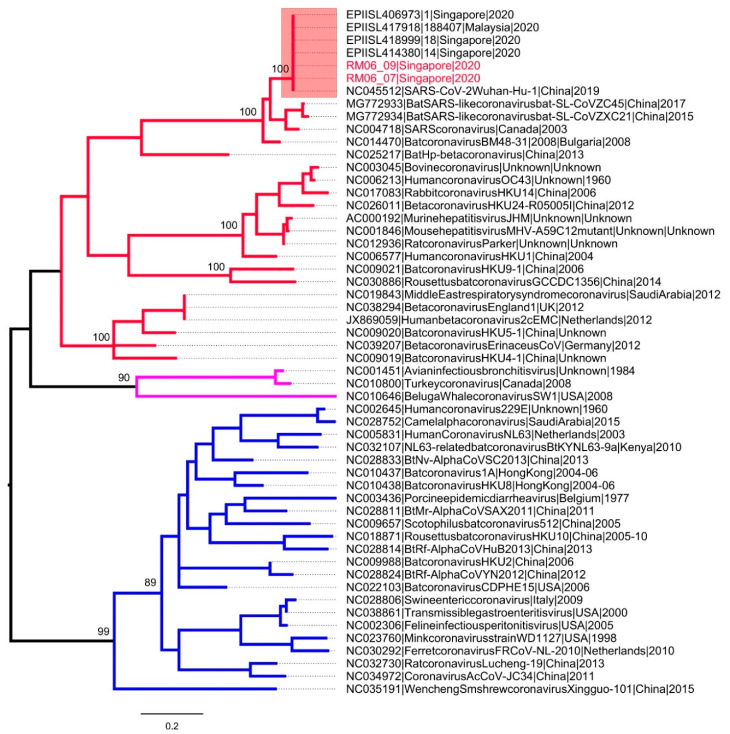
Phylogenetic analysis of RNA dependent RNA polymerase gene (356 bp) of coronaviruses. The maximum likelihood tree was constructed by using the general time reversible substitution model with gamma and invariant site parameters (GTR + Gamma 5 + I). Different groups of coronaviruses are shown in different branch colors; group 1 (blue), group 2 (red) and group 3 (purple). SARS-CoV-2 sequences are highlighted in the pink box. The taxa names of SARS-CoV-2 sequences obtained from two positive swabs are highlighted in red. The numbers shown on nodes are bootstrap values.

Among 428 swab samples taken, only two were positive for SARS-CoV-2 RNA (Table 3). The positive PCR controls confirmed successful amplification in all samples. None of the negative PCR controls yielded positive results, eliminating false positives due to PCR cross-contamination. SARS-CoV-2 was also not detected in field blanks from all surfaces. These positive results were also confirmed by two probe-based real-time PCR assays that targeted different genes of SARS-CoV-2, namely nucleocapsid protein (HKU screening assay; 110 bp) and open reading frame 1b (China CDC confirmatory assay; 132 bp). The positive samples were swabs from the bedside wall and bed handle of the accommodation room of Case 1, before disinfection and cleaning was carried out. Sequencing of the nested PCR fragments (356 bp) yielded identical genetic sequences (Figure 2) and confirmed the environmental contamination by SARS-CoV-2. Both study sequences were identical to SARS-CoV-2 sequences reported earlier in Singapore (Figure 2). The room was cleaned on the same day, and samples collected after the cleaning had undetectable SARS-CoV-2, suggesting that the cleaning methodology was efficacious in lowering the virus load to undetectable levels. The presence of virus contamination on surfaces of a primary space occupied by a case for a prolonged period of time highlights the risk of fomite-mediated transmission and necessitates the regular cleaning and disinfection of indoor and outdoor surfaces, particularly when COVID-19 transmission is present. The risk has been borne out by a report on the epidemiological link between two sets of cases who had not met, but sat in the same seats in sequential services of a church in Singapore [23]. The risk of environmental contamination is corroborated by studies in healthcare settings, including a recent one on SARS-CoV-2 in a Singapore hospital where 60.7% of the samples were found to be positive, and previously on MERS-CoV (20.4%) and SARS-CoV (7.6%) [6,8,10]. The higher contamination rate in hospitals can be attributed to the duration of stay of cases, and the viral shedding capacity of a patient at different stages of the disease.

SARS-CoV-2 was not detected in the majority (99.5%, 426/428) of pre- and post-cleaning samples. The general low positive rate, even before cleaning and disinfection, demonstrates the challenge in detecting SARS-CoV-2 in contaminated environments, especially due to the time lapse between exposure and sampling. As environmental samples often contain low amounts of viral RNA, the assays used for the detection of SARS-CoV-2 need to be highly sensitive. The positive accommodation room was the only site where sampling was performed a day after the case vacated the room after spending a day with symptoms. The small area (4 m^2^) of the affected room might have increased the probability of capturing positive samples. These findings were corroborated by previous evidence of environmental contamination on beddings of quarantine rooms [24], as well as on a cruise ship where SARS-CoV-2 RNA was detected mostly in enclosed cabins and toilets [25].

Although the use of a viral RNA detection method does not always correlate with virus integrity, viability or infectivity [26], a few reports have successfully cultured the virus from environmental and air samples [27]. The fact the virus genetic material could be found in environmental surfaces in our study thus suggests the risk of fomite-mediated transmission.

The negative results in common areas, such as elevators and their call buttons (for Cases 5–6), as well as shared toilets (for Cases 1–3), even before cleaning, could possibly be contributed by the following factors: (i) cases had not dwelled in these areas for a prolonged period of time, and the transient exposure did not lead to a substantial viral load in the environment; (ii) the frequent cleaning regime implemented since Singapore received imported cases had mitigated the risk; (iii) the warm and humid tropical climate has reduced the viral persistence, especially in naturally ventilated spaces. These are perhaps the factors that have led to the contrasting results from the 2003 finding at the Hong Kong Metropole hotel elevator area, where SARS-CoV viral RNA was detected even three months after the case had left the hotel [28]. In addition, SARS-CoV-2 viral RNA was detected on public benches in Brazil [29], but detection of SARS-CoV-2 in that instance could also be explained by its location in a city with a high number of notified COVID-19 cases, and that regular disinfection had not been carried out.

Although laboratory tests have shown that SARS-CoV-2 could be found in aerosols [12], the virus was not detected in any of the air samples in this study. This was expected since premises were vacated at least 24 h prior to sampling. On a similar note, SARS-CoV-2 was not detected in the return or supply air vents of different air-conditioner systems, in contrast to a previous case report in a hospital isolation room [6,8,10]. In the latter case report, high viral shedding from the resident patient, air flow pattern and exhaust outlet location were thought to be possible factors for the virus to be found on the exhaust outlet. Further investigations in non-health care settings is needed for risk assessment.

## 4. Conclusions

The detection of viral RNA in the accommodation room of a confirmed case highlights the importance of disinfection in the community, especially primary spaces of cases, to avoid fomite-mediated transmission. While the study did not reveal gross contamination of the environment, especially secondary sites visited by the cases, high frequency general cleaning of common areas has and will likely mitigate transmission in the community.

## Figures and Tables

**Table 1:** Sampling locations, type of air-ventilation and disinfectants used.

Case	No. of Sites	No. of Samples ^1^	Days after Occupancy	Estimated Floor Area (m^2^)	Ventilation	Air-Conditioning System	Active Ingredient in Disinfectants
1 ^2^	Commercial Boarding Room (n = 1) Toilets (n = 6)	120 ^3^	1	Room—4 Toilet—1.2	Mechanical ventilation limited to toilets only	Wall-mounted fan coil unit	Sodium chlorite
2 ^4^	Home Residence Room (n = 2) Toilet (n = 1)	100	3	Room 1—12 Room 2—14.5 Toilet—3.8	Naturally ventilated, ambient temperature	Wall-mounted fan coil unit (not used since case vacated)	Hydrogen peroxide and peroxyacetic acid
3 ^4^			1				
4	Commercial Boarding Room (n = 1) Toilet (n = 1)	40	3	Room—50 Toilet—5	Mechanical ventilation	Fan coil unit	Benzalkonium chloride
5	Elevators of public housing (n = 3)	96	2	1.7	Mechanical and natural, ambient temperature	Nil	Sodium hypochlorite
6	Elevators of public housing (n = 3)	78	3	1.7	Mechanical and natural, ambient temperature	Nil	Sodium hypochlorite

^1^ Half of the surface swab and air samples were taken before the cleaning and disinfection and the other half was taken after the disinfection procedure, performed by third party commercial companies. ^2^ Case 1 resided in a boarding room with shared toilets. ^3^ Comprises of 114 swab samples and six air samples. ^4^ Cases 2 and 3 used two different rooms in a shared residence.

**Table 2:** Oligonucleotides used in RT-PCR assays.

PCR Round	Primer	Sequence(5′-3′)	Target Region	Product Size
First round	SARSCoV2_FN1	ggttgggattatcctaaatgtga	RNA dependent RNA polymerase (RdRp) gene	440-bp
	SARSCoV2_RN1	gcatcgtcagagagtatcatcat		
Second round /Nested	SARSCoV2-F2N2	ATGCTTAGAATTATGGCCTCACTTG		356 bp
	SARSCoV2_RN2	CGTAAAACTCATTCACAAAGTCTGT		

**Table 3:** Environmental samples and corresponding nested RT-PCR results.

Sampling Location and Surface	RT-PCR Results in Samples Collected before and after Disinfection ^1^					
	Before	After	Before	After	Before	After
	Case 1		Cases 2 and 3		Case 4	
**Accommodation room**						
Air samples	0 (3)	0 (3)	NA	NA	NA	NA
Air-conditioner filters and vents	0 (6)	0 (6)	0 (6)	0 (6)	0 (1)	0 (1)
Bed handle	1 (1)	0 (1)	NA	NA	NA	NA
Bed headboards	1 (1)	0 (1)	0 (2)	0 (2)	0 (1)	0 (1)
Beside walls	0 (2)	0 (2)	0 (2)	0 (2)	NA	NA
Chairs/ arm rests	0 (1)	0 (1)	0 (2)	0 (2)	0 (1)	0 (1)
Door handles	0 (1)	0 (1)	0 (2)	0 (2)	0 (1)	0 (1)
Floor	NA	NA	0 (2)	0 (2)	NA	NA
Cups	NA	NA	0 (2)	0 (2)	0 (1)	0 (1)
Travel items	NA	NA	0 (2)	0 (2)	0 (1)	0 (1)
Phone	NA	NA	NA	NA	0 (1)	0 (1)
Television remote and computer controls	NA	NA	0 (6)	0 (6)	0 (1)	0 (1)
Safe buttons	NA	NA	NA	NA	0 (1)	0 (1)
Switches	0 (2)	0 (2)	0 (2)	0 (2)	0 (2)	0 (2)
Tables	0 (1)	0 (1)	0 (2)	0 (2)	0 (2)	0 (2)
Wardrobe	NA	NA	0 (2)	0 (2)	NA	NA
Window panels and handles	NA	NA	0 (7)	0 (7)	NA	NA
Sub Total	2 (18)	0 (18)	0 (39)	0 (39)	0 (13)	0 (13)
**Toilets**						
Exhaust vents	0 (6)	0 (6)	NA	NA	NA	NA
Door handle	0 (6)	0 (6)	0 (2)	0 (2)	NA	NA
Shower door handle	0 (6)	0 (6)	0 (1)	0 (1)	0 (1)	0 (1)
Sink rims	0 (6)	0 (6)	0 (2)	0 (2)	0 (1)	0 (1)
Sink taps	0 (6)	0 (6)	0 (1)	0 (1)	0 (1)	0 (1)
Toilet bowl/rim	0 (6)	0 (6)	0 (2)	0 (2)	0 (1)	0 (1)
Toilet flush	0 (6)	0 (6)	0 (1)	0 (1)	0 (1)	0 (1)
Toilet seat	NA	NA	0 (2)	0 (2)	0 (2)	0 (2)
Sub Total	0 (42)	0 (42)	0 (11)	0 (11)	0 (7)	0 (7)
**Elevators**	**Case 5**		**Case 6**			
Elevator lobby buttons	0 (6)	0 (6)	0 (6)	0 (6)		
Exterior elevator doors	0 (6)	0 (6)	0 (6)	0 (6)		
Interior elevator walls	0 (9)	0 (9)	0 (15)	0 (15)		
Interior elevator handles	0 (9)	0 (9)	0 (9)	0 (9)		
Interior elevator doors	0 (3)	0 (3)	0 (3)	0 (3)		
Elevator floors	0 (3)	0 (3)	0 (3)	0 (3)		
Elevator buttons	0 (3)	0 (3)	0 (6)	0 (6)		
Sub Total	0 (39)	0 (39)	0 (48)	0 (48)		

^1^ Number of samples tested is given in brackets. Number of RT-PCR positive samples among the tested is given outside the brackets. NA = Not available.

## Data Availability

Sequences of SARS-CoV-2 generated in the present study were deposited in Genbank database under the accession numbers MT309050 and MT309051.

## Supplementary Materials

The following are available online at https://www.mdpi.com/1660-4601/18/1/117/s1, Table S1. Sensitivity of different PCR assays for the detection of SARS-CoV-2; Table S2. The details and list of authors of the sequences retrieved from GISAID’s EpiCoV database.
