# Supplementary material for: Environmental Contamination of SARS-CoV-2 in a Non-Healthcare Setting
Source: Int J Environ Res Public Health. 2020 Dec 26;18(1):117. doi: 10.3390/ijerph18010117 (PMC7797951; doi:10.3390/ijerph18010117)
Supplement: Supplementary file 1 [file ijerph-18-00117-s001.pdf]

Table 1. Sensitivity of different PCR assays for the detection of SARS-CoV-2**.**

RNA copies	Assay			
	HKU-N assay	HKU-ORF assay	China CDC assay	Conventional nested PCR
100000	26.33	28.18	21.94	Positive
5000	31.31	33.11	26.59	Positive
250	35.77	36.99	31.45	Positive
12.5	Negative	Negative	36.03	Positive
0.6	Negative	Negative	Negative	Negative

Hong Kong University (HKU) assays target the nucleocapsid protein (screening assay) and Open Reading Frame (ORF) 1b. China CDC assay targets ORF 1ab. All real-time PCR assays were evaluated by using the Luna Universal qPCR Master Mix (New England Biolabs, USA), according to the protocols recommended by the developers of respective assays. The conventional nested PCR assay targets the RNA dependent RNA polymerase gene. All assay evaluations were done using 2 μL of template dilutions. The dilutions were obtained from a synthetic RNA control of SARS-CoV-2 with known copy numbers (1+E6 copies per μL; Twist Bioscience, USA). The detection threshold for all real-time PCR assays was $Cq < 40$.

Table S2. The details and list of authors of the sequences retrieved from GISAID's EpiCoV database

Accession ID	Virus name	Location	Collection date	Originating lab	Submitting lab	Authors
EPI_ISL_410537	hCoV-19/Singapore/6/2020	Asia / Singapore	2020-02-09	Singapore General Hospital, Molecular Laboratory, Division of Pathology	Programme in Emerging Infectious Diseases, Duke-NUS Medical School	Danielle E Anderson, Martin Linster, Yan Zhuang, Jayanthi Jayakumar, Kian Sing Chan, Lynette LE Oon, Shirin Kalimuddin, Jenny GH Low, Yvonne CF Su, Gavin JD Smith
EPI_ISL_410536	hCoV-19/Singapore/5/2020	Asia / Singapore	2020-02-06	Singapore General Hospital, Molecular Laboratory, Division of Pathology	Programme in Emerging Infectious Diseases, Duke-NUS Medical School	Danielle E Anderson, Martin Linster, Yan Zhuang, Jayanthi Jayakumar, Kian Sing Chan, Lynette LE Oon, Shirin Kalimuddin, Jenny GH Low, Yvonne CF Su, Gavin JD Smith
EPI_ISL_406973	hCoV-19/Singapore/1/2020	Asia / Singapore	2020-01-23	Singapore General Hospital	National Public Health Laboratory	Mak, TM; Octavia S; Chavatte JM; Zhou, ZY; Cui, L; Lin, RTP
